# Design of short peptides to block BTLA/HVEM interactions for promoting anticancer T-cell responses

**DOI:** 10.1371/journal.pone.0179201

**Published:** 2017-06-08

**Authors:** Marta Spodzieja, Sławomir Lach, Justyna Iwaszkiewicz, Valérie Cesson, Katarzyna Kalejta, Daniel Olive, Olivier Michielin, Daniel E. Speiser, Vincent Zoete, Laurent Derré, Sylwia Rodziewicz-Motowidło

**Affiliations:** 1 University of Gdansk, Department of Chemistry, Gdansk, Poland; 2 SIB Swiss Institute of Bioinformatics, Quartier Sorge, Batiment Genopode, Lausanne, Switzerland; 3 Urology Research Unit, Urology Department, University Hospital of Lausanne (CHUV), Lausanne, Switzerland; 4 Team Immunity and Cancer, Centre de Recherche en Cancérologie de Marseille (CRCM), Inserm U1068, CNRS, UMR7258, Institut Paoli-Calmettes, Aix-Marseille University, Marseille, France; 5 Ludwig Center for Cancer Research of the University of Lausanne, Epalinges, Switzerland; 6 Department of Oncology, University of Lausanne and University Hospital of Lausanne (CHUV), Lausanne, Switzerland; 7 Department of Oncology, Ludwig Cancer Research Center, Epalinges, Switzerland; 8 Department of Fundamental Oncology, University of Lausanne, Epalinges, Switzerland; University of Cambridge, UNITED KINGDOM

## Abstract

Antibody based immune-checkpoint blockade therapy is a major breakthrough in oncology, leading to clinical benefit for cancer patients. Among the growing family of inhibitory receptors, the B and T lymphocyte attenuator (BTLA), which interacts with herpes virus entry mediator (HVEM), is a promising target for immunotherapy. Indeed, BTLA inhibits T-cell proliferation and cytokine production. The crystal structure of the BTLA/HVEM complex has shown that the HVEM(26–38) fragment is directly involved in protein binding. We designed and analyzed the capacity of several analogs of this fragment to block the ligation between BTLA and HVEM, using competitive ELISA and cellular assay. We found that the HVEM(23–39) peptide can block BTLA/HVEM ligation. However, the blocking ability was due to the Cys encompassed in this peptide and that even free cysteine targeted the BTLA protein and blocked its interaction with HVEM. These data highlight a Cys-related artefact *in vitro*, which should be taken in consideration for future development of BTLA/HVEM blocking compounds.

## Introduction

T lymphocytes are central players of the adaptive anti-tumor immune response. Activation of T lymphocytes requires two signals: the first one is originating from the T-cell receptor after interaction with an antigen-derived peptide presented by major histocompatibility complex, while the second is delivered upon interactions between co-receptors on T cells and their ligands on antigen-presenting cells (APCs) or target cells [1]. Co-receptors, also called immune checkpoints, can be divided into stimulatory and inhibitory molecules. The CD28 was the first identified co-stimulatory molecule and the interaction between CD28 and B7.1/B7.2 is one of the dominant pathways required for full activation of naive lymphocytes [2, 3]. In contrast, cytotoxic T-lymphocyte-associated protein 4 (CTLA-4) and programmed cell death 1 protein (PD-1), when interacting with their ligands B7.1/B7.2 and PDL-1/PDL-2 respectively, restrain the activation of lymphocytes T and are thus considered as co-inhibitory molecules [4–7].

It is known that manipulation of checkpoint cell-surface signaling molecules can exert potent anticancer effects. Immune checkpoint therapy has recently reached important clinical advances [8, 9]. Indeed, it has been reported that cancer patients treated with anti-CTLA-4 (*Ipilimumab*) or anti-PD-1 (*Nivolumab* or *Pembrolizumab*) blocking antibodies showed an increase of the antitumor T-cell response and a higher rate of disease free survival [10–13]. Additionally, antibodies blocking PD-L1 were shown to be superior to *Ipilimumab* by having a better safety profile. Therapies combining both *Ipilimumab* and *Nivolumab* in patients with metastatic melanoma, have shown significant increase of the progression-free survival rate. However, this combination was also associated with substantial immune related toxicities [12, 14–17].

Alternative non-antibody based therapies constitute also a promising approach to target immune checkpoints [18, 19]. Indeed, several examples of small compounds such as sulphonamide derivatives [20], tri-aromatic structures [18, 21], linear, cyclic macrocyclic peptides [18, 22], hydrolysis-resistant D-peptide [23], peptidomimetics and cyclic peptidomimetics [22], as antagonist of PD-1/PD-L1 have already been reported. Some of these compounds are highly effective in antagonizing PD-1 signaling and inhibiting tumor growth and metastasis in preclinical models of cancer. Moreover, those compounds are well tolerated with no obvious toxicity [15, 18].

Among the growing family of inhibitory receptors, the B and T lymphocyte attenuator (BTLA), which interacts with herpes virus entry mediator (HVEM), inhibits T-cell proliferation and cytokine production [24, 25], suggesting that BTLA might be an interesting target in immunotherapy [26]. Naive human CD8^+^ T cells express high levels of BTLA, which is downregulated upon CD8 differentiation. However, BTLA is not downregulated in melanoma specific CD8^+^ T cells and remains susceptible to functional inhibition upon HVEM ligation. HVEM is frequently expressed on melanoma cells, suggesting that the BTLA/HVEM pathway might play a role in the inhibition of efficient immune responses against cancer. Preventing/targeting the BTLA/HVEM interaction can reverse the inhibitory functions of BTLA, thus triggering the immune response against cancer. Additionally, *in vitro* studies using antibodies to block binding of BTLA to HVEM led to increased melanoma specific CD8^+^ effector T-cells proliferation and enhanced cytokines production [27–29].

The crystal structure of BTLA/HVEM complex shows specific details of the proteins interaction. BTLA binds to a fragment of HVEM(26–33) which is located in the Cysteine Rich Domain 1 (CRD1) [30]. We used that information to design and characterize peptide-based inhibitors of BTLA/HVEM complex formation. We confirmed that the short fragment of HVEM(23–39) binds to BTLA and blocks the BTLA/HVEM interactions. Further evaluation of individual contribution of each residue from the HVEM(23–39) fragment by a single alanine substitution approach showed that cysteine residues have a key role in the binding to BTLA. Finally, we showed that free cysteine amino acid can disrupt the BTLA/HVEM complex formation, highlighting a cysteine-related artefact *in vitro*. Overall, these results might be helpful for the design of new compounds targeting BTLA protein.

## Materials and methods

### Recombinant molecules and antibodies

The recombinant human BTLA protein used in affinity tests was purchased form Novoprotein (Company product code: C563). For ELISA, recombinant human BTLA-Fc, HVEM-Fc as well as anti-human BTLA (#7.1) blocking monoclonal antibody (mAb) and anti-human HVEM (#11.8) non-blocking mAb were described previously [31]. Anti-HVEM mAb was biotinylated using the Biotin Labeling Kit-NH2 (Abnova, #KA0003), according manufacturer’s instruction.

### Molecular modeling

The crystal structure of BTLA/HVEM complex (A and B chains) served as a starting point for the modeling (Protein Data Bank ID 2AW2) [30]. The atoms of the HVEM B chain were removed, except for residues 23–39. The protonation state of the histidines was set depending on the environment, to optimize hydrogen bonds. All simulations were carried out using GROMACS 4.5 molecular modeling package [32] using the CHARMM22 force field [33]. The structure was located in the center of a TIP3P water box in which the distance between the solute and the edges was 1.4 nm. Na^+^ and Cl^-^ ions were added to neutralize the system. Subsequently the system was subjected to 1000 steps of steepest descent minimization. The structures were then equilibrated according to standard equilibration procedures. The system heating was performed using simulated annealing method by increasing the temperature in four steps: from 0K to 50K, then from 50K to 100K, from100K to 200K and finally from 200K to 300K. Every step took 100ps and at the end of the procedure the system was additionally coupled to the target 300K temperature for 100ps. The force constant of 1000kJ/mol*nm was applied to keep the atoms in their positions during the heating procedure. The same force constant was kept also during the first constant volume equilibration simulation during 100ps, followed by another 100ps simulation in the NVT scheme with lowered force constant for position restraint to 300kJ/mol*nm. The final step of equilibration consisted in a simulation in the NPT scheme with Nose-Hoover thermostat and Parinello-Rahman barostat with 300K and 1Pa for 500ps and no position restraints imposed on atoms.

The production molecular dynamics simulations were done using the NPT scheme with no restraints on the atoms. For the production molecular dynamics simulation, the Verlet leap frog integrator was used. A 12Å cutoff was applied on non-bonded interactions. The bond length was constraint. A time step of 2fs was used with a length of the simulation of 10ns. For the hydrogen bonds analysis, we used 250 structures extracted every 40ps from the molecular dynamics simulation trajectory.

Molecular graphics images were produced using the UCSF Chimera package from the Computer Graphics Laboratory, University of California, San Francisco (supported by NIH P41 RR-01081) [34].

### Peptide synthesis

Peptides were synthesized by solid phase peptide synthesis (SPPS) using semiautomated peptide synthesizer Millipore 9050 Plus PepSynthesizer (Millipore Corporation, Burlington, VT, USA) and general conditions of solid-phase synthesis [35]. Synthesis was performed on a TentaGel R RAM resin (0.19 mmol/g), using 9-fluorenylmethoxycarbonyl/tert-butyl (Fmoc/tBu) chemistry with the following side chain protected amino acid derivatives: Fmoc-Pro-OH, Fmoc-Glu(OtBu)-OH, Fmoc-Cys(Trt)-OH, Fmoc-Val-OH, Fmoc-Ala-OH, Fmoc-Gly-OH, Fmoc-Thr(tBu)-OH, Fmoc-Leu-OH, Fmoc-Lys(Boc)-OH, Fmoc-Arg(Pbf)-OH and Fmoc-Tyr(tBu)-OH. Acetylation of the *N*-terminal amino group was performed using 1-acetylimidazole (1.10 g/1 g of resin at room temperature for 24 h). The peptide was cleaved from the resin for 2 h using a mixture of 88% TFA, 5% fenol, 5% deionized water and 2% triisopropylsilane (10 ml/1 g of resin at room temperature for 2 h). After filtration of the exhausted resin, solution was concentrated *in vacuum*, and the residue was triturated with Et_2_O. The precipitated peptide was centrifuged for 15 min, 4000 rpm, followed by decantation of the ether phase from the crude peptide (process was repeated three times). After evaporation of Et_2_O, the peptide was dissolved in H_2_O and lyophilized.

### Peptide purifications

Before the purification the peptides were dissolved in H_2_O, DTT was added (6-fold excess with respect to purified peptide) and the mixture was sonified for 30 min in 60°C. Purification of the crude peptide was carried out by using RP-HPLC on a semi-preparative Phenomenex Luna C8(2) (250 mm x 20 mm, 5 μm) column. A linear gradient from 5% B to 50% B in A in 150 min was used. The aqueous system (A) consisted of 0.1% (v/v) TFA solution in water, whereas the organic phase (B) was 80% acetonitrile in water, containing 0.08% (v/v) TFA. Purification was monitored by UV absorption at a wavelength of 222 and 254 nm. The purity of the peptide was verified by LC-ESI-IT-TOF/MS (Shimadzu, Shimpol, Warsaw, Poland), and by using RP-HPLC with a Kromasil C8 analytical column (250mm x 4.6 mm, 5 μm), where a gradient of 5% to 100% B in A in 60 min was employed, with A and B as described above.

### Disulfide bond formation

The formation of the disulfide bond in peptide HVEM(23–39) was performed after purification. The peptide was dissolved in a mixture of H_2_O and methanol (1:9, v:v), the final volume was 1.5 l and the concentration of the peptide was established at 40 mg/l. The pH was adjusted and kept between 8 and 9 by using ammonia whilst stirring the solution at room temperature for 3 days in the presence of atmospheric oxygen. Reaction progress was monitored by analytical RP-HPLC in a gradient of 5% to 100% B in A in 60 min. When the reaction of oxidation was completed the solvents were evaporated and the peptide was lyophilized.

### ELISA

96-well round bottom plates (medium binding; Greiner Bio-One) were coated with 400 ng/well of recombinant BTLA-Fc protein in PBS (50 μL/well) and incubated overnight at 4°C. Wells were washed twice with PBS-T (phosphate buffer saline with 0.1% Tween-20, pH 7.4) and blocked with 1% milk in PBS-T for 1 hour at 37°C. After 2 washes with PBS-T, the peptides were titrated down in triplicates from 10 to 0.1 mg/well in 1% milk/PBS-T and incubated for 2 hours at 37°C. Maximum binding of HVEM to BTLA was controlled by adding 1% milk/PBS-T to negative control wells, whereas maximum inhibition was monitored by adding anti-BTLA 7.1 mAb (0.1 μg/well). Next, the plate was washed and incubated with recombinant HVEM-Fc protein at 400 ng/well in 1% milk/PBS-T for 1 hour at 37°C. Biotinylated anti-HVEM 11.8 antibody was then added (biotinylated preparation diluted at 1/10 000 in 1% milk/PBS-T) and incubated for 1 hour at 37°C. Streptavidin horseradish peroxidase (Dako) was finally added at a 1/8000 dilution in 1% BSA/PBS-T for 30 min at 37°C and detected with TMB (Biorad). The substrate reaction was blocked with 0.2 M H_2_SO_4_ and the absorbance was read at 450 nm and 630 nm. The percent of inhibition was calculated assuming that the anti-BTLA 7.1 antibody inhibit the complex at 100% and milk/PBS-T in 0%. Statistical analysis of the results was performed using the one-way analysis of variance (one-way ANOVA).

### Preparation of microcolumn

The immobilization of BTLA protein in a microcolumn was performed using the NHS-activated acid-coupled Sepharose 4B (Sigma Aldrich). BTLA protein was dissolved in 100 μl of coupling buffer (0.2 M NaHCO_3_, 0.5 M NaCl, pH 8.3) and was added to 0.06 g NHS-activated Sepharose in 100 μl of coupling buffer. The coupling reaction was performed for 1 hour in 25°C and the mixture was transferred into the microcolumn (MoBiTec, Goettingen, Germany). The microcolumn with immobilized protein was washed alternately with 10 ml of solution blocking buffer (0.1 M amino ethanol, 0.5 M NaCl, pH 8.3) and washing buffer (0.1 M CH_3_COONa, 0.5 M NaCl, pH 4). This step was repeated 4 times. The microcolumn was stored in PBS (pH 7.4).

### Affinity tests

Peptide affinity assay using NHS-activated Sepharose microcolumn was already described elsewhere [36, 37]. The peptides were added onto BTLA-Sepharose column equilibrated in ammonium hydrogen carbonate (NH_4_HCO_3_, pH 7.4) and incubated for 2 h at 25°C with gentle shaking. After this time the unbound peptide was removed and the column was washed with 100 ml NH_4_HCO_3_ and 20 ml of H_2_O. Dissociation of the protein—peptide complex was performed using 0.1% TFA in H_2_O (2 x 0.5 ml) by 15 minutes. All fractions were analyzed using mass spectrometry (MALDI TOF/TOF^™^ 5800, AB SCIEX).

### Ellman’s assay

The presence of free thiols was investigated using Ellman’s assay. DTNB (Sigma Aldrich) was first dissolved in reaction buffer (0.1 M sodium phosphate, pH 7.4, containing 1 mM EDTA) at a concentration of 4 mg/ml. Then peptides and recombinant BTLA-Fc, alone or mixed together (ratio 1:1) were added in DTNB reaction buffer. After 15 minutes of incubation, the SH content of protein samples was then assessed by the measurement of absorbance at 412 nm. For each sample, the absorbance value from the blank was subtracted.

### BTLA/HVEM interaction—Cell line assay

293T cells expressing BTLA (generous gift from Dr. C. Krummenacher, University of Pennsylvania, USA) were maintained in culture with RPMI 10% FCS supplemented with 0.2 mg/ml hygromycin B. 2x10^5^ 293T cells expressing BTLA were incubated with rhHVEM-Fc at 8μg/ml in PBS 0.5% BSA for 30min at 4°C. Cells were then washed and incubated with goat anti-human Fc conjugated with AF647 for 20min at 4°C (Jackson ImmunoResearch, #109-606-170). Inhibition of HVEM-BTLA binding by different peptides was assessed by pre-incubating the cells with the different peptides at 5 mg/ml for 1h at 4°C. BTLA expression was controlled by anti-BTLA APC-conjugated antibody (Biolegend, #344510). Maximum inhibition of binding was determined by adding anti-BTLA7.1 antibody for 1h at 4°C at 2μg/ml before adding rhHVEM-Fc. Sample acquisition was performed on the Gallios Flow-Cytometer (Beckman Coulter) and data were analyzed using the FlowJo Software (TreeStar).

## Results

### Design of peptides targeting BTLA protein

**Fig 1:**
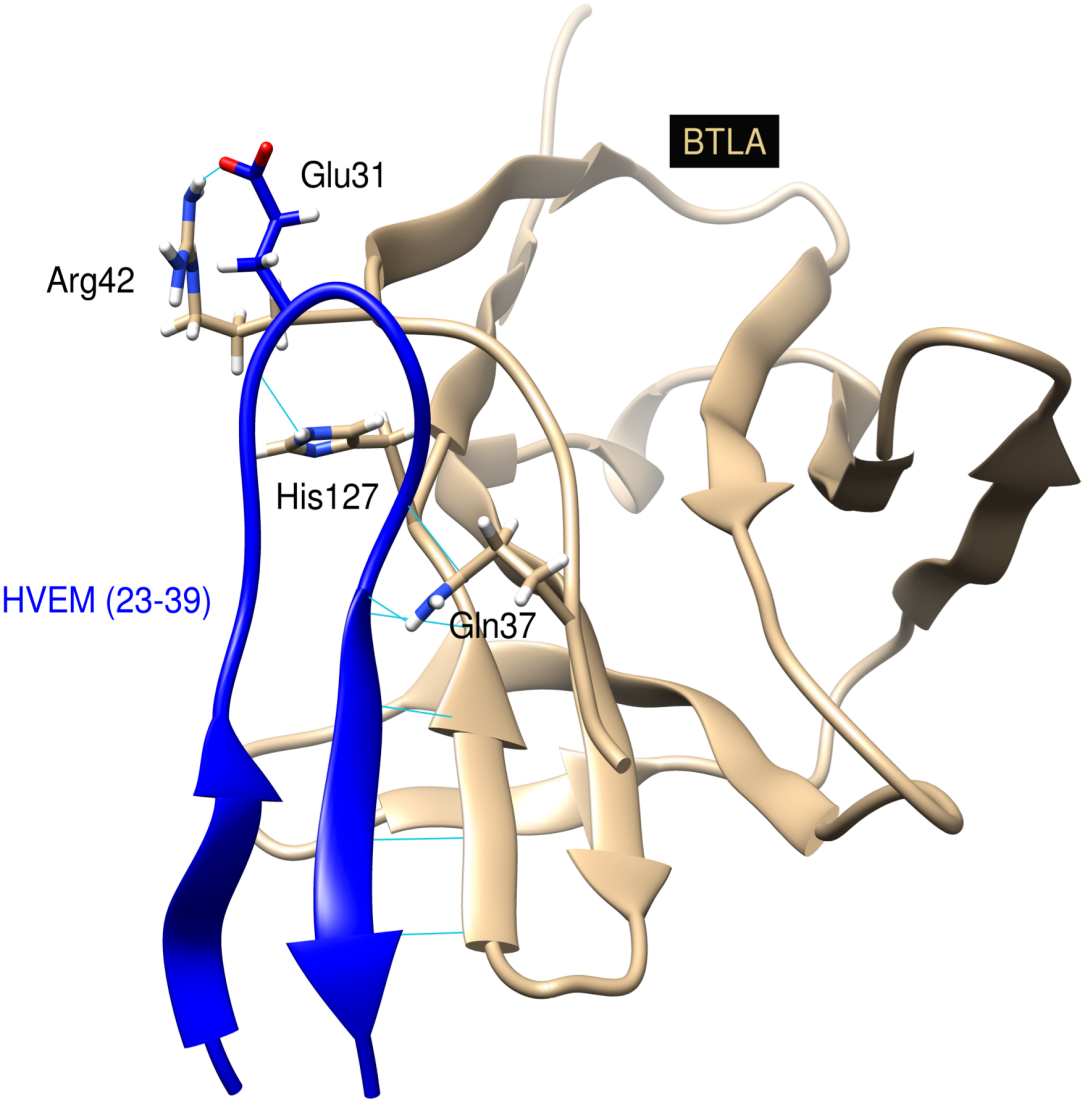
Structure of the BTLA/HVEM(23–39) peptide complex. HVEM(23–39) peptide is shown in dark blue ribbon, BTLA in beige and the side chains of residues involved in the inter-molecular hydrogen bonds in stick representation with nitrogen atoms colored in blue, oxygen colored in red and hydrogen atoms in white. Hydrogen bonds are represented by light blue lines.

The crystal structure of BTLA/HVEM complex is stored under ID 2AW2 in the Protein Data Bank [30]. The structure indicates that only the first CRD1 part of human HVEM interacts with BTLA. The domain contains four short β-strands, that are connected by three disulfide bonds, formed between cysteine residues 4–15, 16–29 and 19–37. The fragment of the CRD1 domain of HVEM comprising residues 23–39 forms a β-hairpin structure and the 35–39 fragment builds an anti-parallel inter-molecular β-sheet with G° strand of BTLA in the center of the interaction interface [30]. Moreover, the HVEM(23–39) fragment comprises two hot-spots of the BTLA/HVEM interaction: Tyr23 and Val36. We sought to determine whether the HVEM(23–39) fragment could bind BTLA. We therefore performed the molecular dynamics simulation of the BTLA structure and the HVEM(23–39) fragment (Ac-YRVKEACGELTGTVCEP-NH_2_). The simulation starting point was a native-like conformation of HVEM fragment, bound to BTLA as in the BTLA/HVEM complex structure (Fig 1). The complex was stable during the 10ns long molecular dynamics simulation. The averaged RMSD calculated for the backbone atoms of the superimposed over carbons alpha structures was 1.2Å. Besides, the hydrogen bonds interactions observed in the starting structure were almost all preserved during the simulation (S1 Table). This computational results suggests that the HVEM(23–39) may stably interact with BTLA.

### Study of the interactions between BTLA protein and HVEM(23–39) fragment

**Fig 2:**
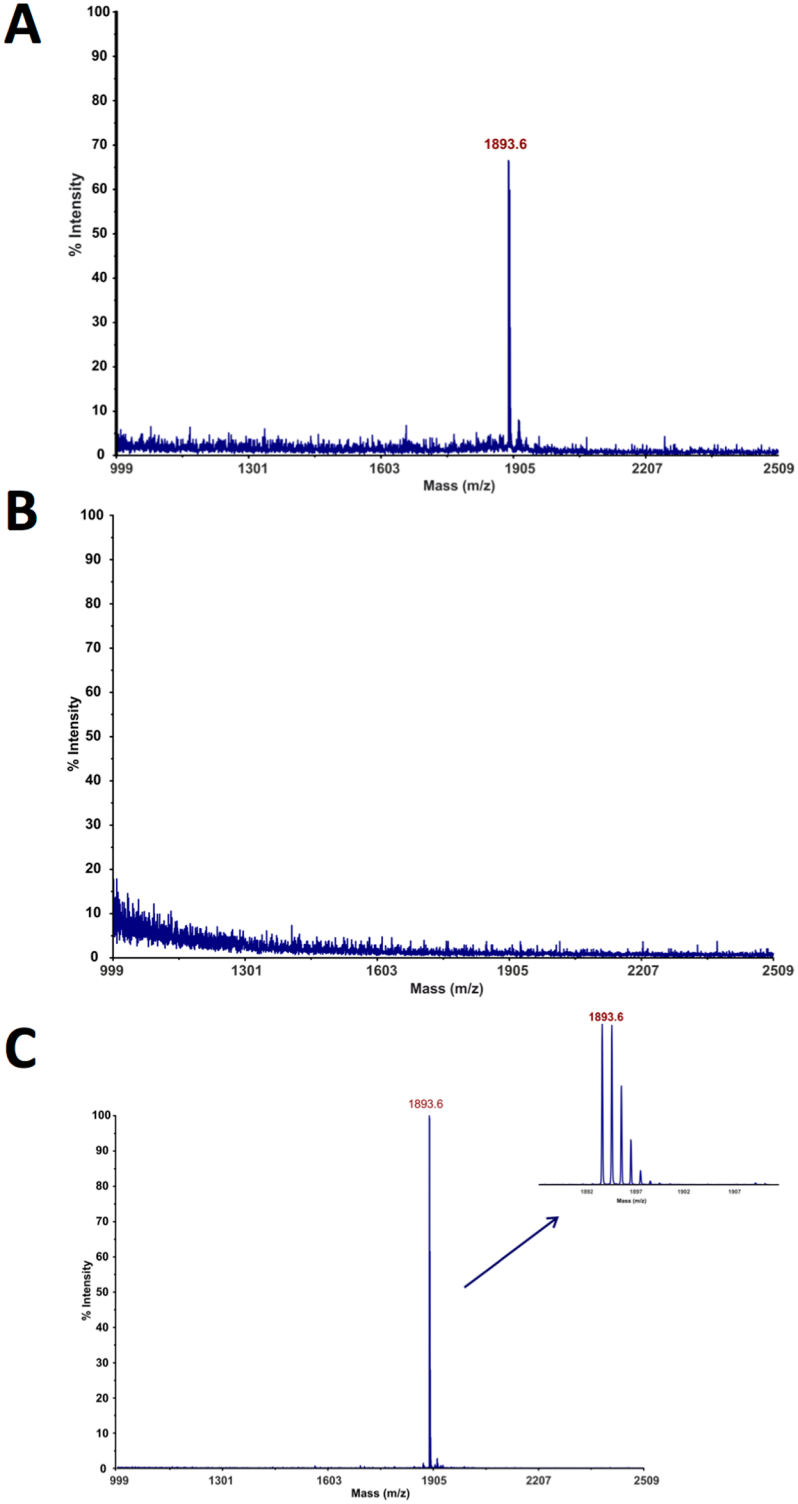
Microcolumn affinity test. Microcolumn affinity test results for binding of HVEM(23–39) fragment to BTLA: (**A)** supernatant, (**B)** last wash and (**C)** elution fractions. The MS measurements were done with the use of MALDI TOF/TOF 5800 (ABSciex, Germany). As a matrix α-cyano-4-hydroxycinnamic acid (CHCA, 10 mg/ml, Sigma-Aldrich) was used. The measurements were done in reflector positive mass mode with previous mass calibration with commercial standard peptide mixture (The Peptide Mass Standards Kit for Calibration of AB SCIEX MALDI-TOF^™^ Instruments).

The designed HVEM(23–39) peptide was synthesized and an affinity test was performed using microcolumn with the immobilized BTLA protein. Three fractions were analyzed: supernatant, last wash and elution by using mass spectrometry techniques. In the supernatant fraction the signal m/z corresponding to the excess of the HVEM(23–39) peptide was observed (Fig 2A). In the last wash fraction, no signal was observed confirming that the excess of peptide was removed (Fig 2B). The presence of m/z signal in elution fraction indicated that the complex between BTLA and HVEM(23–39) was formed (Fig 2C).

**Fig 3:**
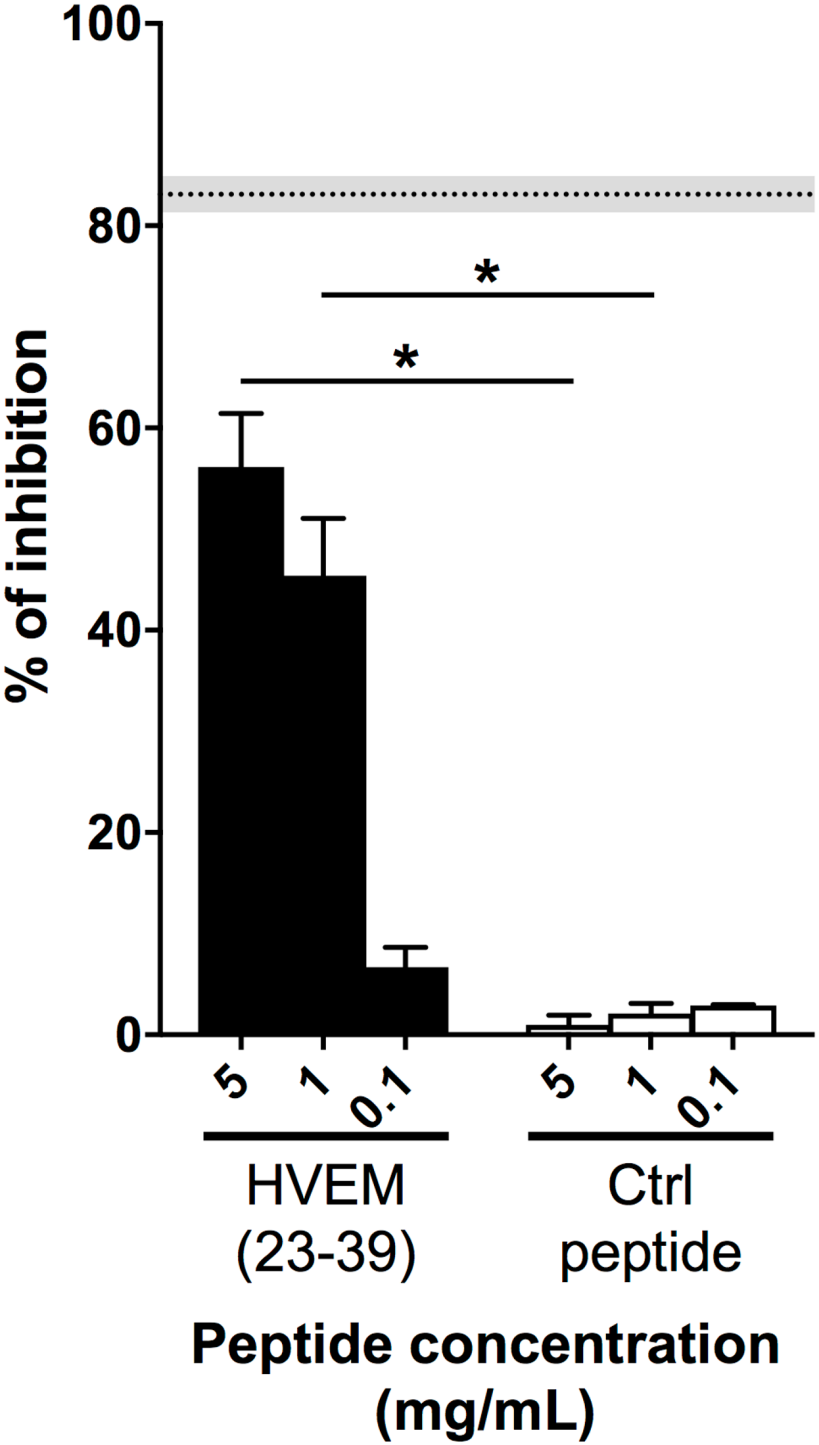
HVEM(23–39) peptide partially blocks the BTLA/HVEM binding. Inhibition of the BTLA/HVEM interaction was assessed by ELISA (at least two experiments in triplicate). The graph shows percentages of inhibition of the BTLA/HVEM ligation, relative to the negative control (PBS), in the presence of different concentrations (5, 1 and 0.1 mg/mL) of HVEM(23–39) and corresponding scrambled peptide (Ctrl peptide). The gray and dotted back lines correspond to the percentages of inhibition observed with an anti-BTLA blocking antibody (Mean +/-SEM). *: p< 0.05 following non-parametric One-way ANOVA and Dunn’s post-test.

Next, HVEM(23–39) was analyzed in an competitive ELISA test, in order to determine whether this fragment may bind to BTLA and inhibit BTLA/HVEM interaction. Fig 3 shows that the HVEM(23–39) can partially prevent the ligation of HVEM to BTLA, in a dose dependent manner, while scrambled peptide (Ac-ELCAGPVTRKVECTYGE-NH_2_) has no effect. Moreover, the scrambled peptide (named Ctrl peptide in figures) did not interact with BTLA immobilized in microcolumn (Table 1).

**Table 1:** Microcolumn affinity test.

Peptides	Supernatant [M+H]^+1^	Last wash	Elution [M+H]^+1^	M_theoric_
HVEM(23–39)	1893.6	-	1893.6	1894.9
HVEM(23–39) C29S	1879.7	-	1879.7	1880.1
HVEM(23–39) C29Y	1955.7	-	1955.7	1956.2
HVEM(31–39)	989.5	-	989.3	988.4
HVEM(31–39) C37S	995.5 [M+Na]	-	-	973.0
Scrambled peptide	1893.6	-	-	1894.9
HVEM(23–39) (C29-C37)	1893.9	-	1893.9	1892.9

### Assessment of amino acid residues from HVEM(23–39) peptide crucial for inhibition of the BTLA/HVEM complex formation

**Fig 4:**
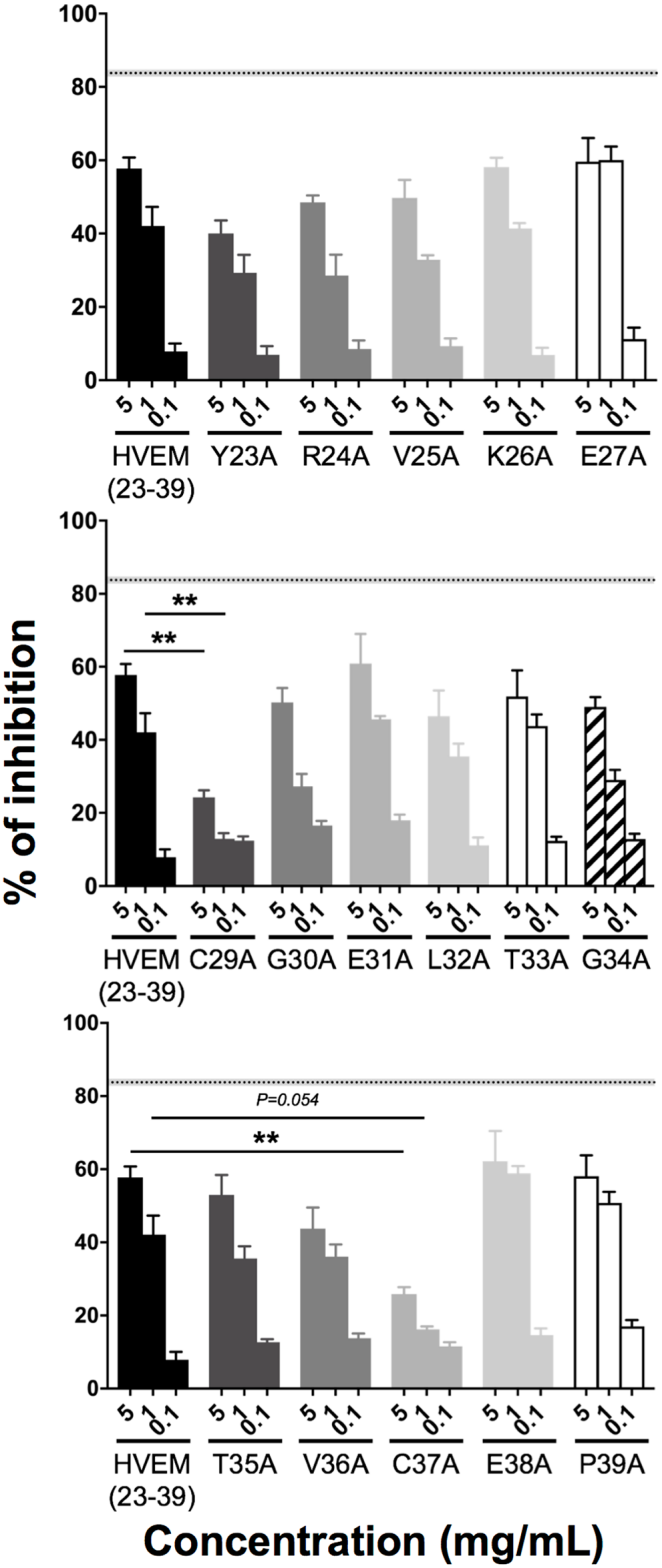
Contribution of individual HVEM(23–39) amino acid residues to the inhibition of BTLA/HVEM interaction. The relative involvement of alanine-substituted HVEM(23–39) peptide analogs was assessed by ELISA (at least two experiments in triplicate) in the presence of graded peptide concentrations (5, 1 and 0.1 mg/mL). The percentage of inhibition of the BTLA/HVEM binding was calculated in relation to the negative control (PBS). The gray and dotted back lines correspond to the percentages of inhibition observed with an anti-BTLA blocking antibody (Mean +/-SEM). Statistical analysis was performed by comparing each concentration of modified peptide to the same concentration of wild-type peptide HVEM(23–39). **: p< 0.01 following non-parametric One-way ANOVA and Dunn’s post-test.

To assess the importance of each amino acid residues in HVEM(23–39) interaction with BTLA protein, single alanine-substituted HVEM(23–39) variants were synthesized [38, 39] and tested in the BTLA/HVEM ELISA. While no or minor diminution in the inhibition of BTLA/HVEM binding was found with the majority of alanine-substituted fragments compared to the native HVEM(23–39), we observed a significant reduction of the inhibition, in a dose depend manner, for the two peptides where cysteine in position 29 (C29A) or 37 (C37A) was replaced by an alanine (Fig 4). This suggests the possible importance of cysteine residues in inhibition BTLA/HVEM complex formation by the HVEM(23–39) peptide.

### The role of cysteine residues in targeting BTLA protein

To further determine the role of cysteine residues from HVEM(23–39) peptide in the inhibition of BTLA/VEM interaction, several HVEM(23–39) analogues were designed and synthesized (Table 2). Initially, we aimed to test, if the Cys29 residue could be replaced with aromatic residue that could interact with His127 residue of BTLA to improve the interaction of the peptide. To test this, we synthesized the HVEM(23–39) C29Y peptide. To additionally probe the importance of the cysteine interactions, we replaced the Cys29 residue with the serine residue, which is of similar size and polarity. We assumed that the C29S analog could mimic the cysteine interactions but bears no free sulfhydryl group that could interact non-specifically with BTLA. We were also wondering, if even shorter HVEM-derived peptide could inhibit the interactions between BTLA and HVEM. We synthesized therefore 9 amino acid-long peptides comprising the ^®^-strand interface of HVEM, HVEM (31–39) fragment.

**Table 2:** Sequences of tested peptides.

Peptides	Amino acid sequence
HVEM(23–39)	Ac-YRVKEACGELTGTVCEP-NH_2_
Scrambled peptide	Ac-ELCAGPVTRKVECTYGE-NH_2_
HVEM(23–39) C29S	Ac-YRVKEA**S**^**29**^GELTGTVCEP-NH_2_
HVEM(23–39) C29Y	Ac-YRVKEA**Y**^**29**^GELTGTVCEP-NH_2_
HVEM(31–39)	Ac-ELTGTVCEP-NH_2_
HVEM(31–39) C37S	Ac-ELTGTV**S**^**37**^EP-NH_2_
HVEM(23–39) (C29-C37)	Ac-YRVKEA**C**GELTGTV**C**EP-NH_2_

**Fig 5:**
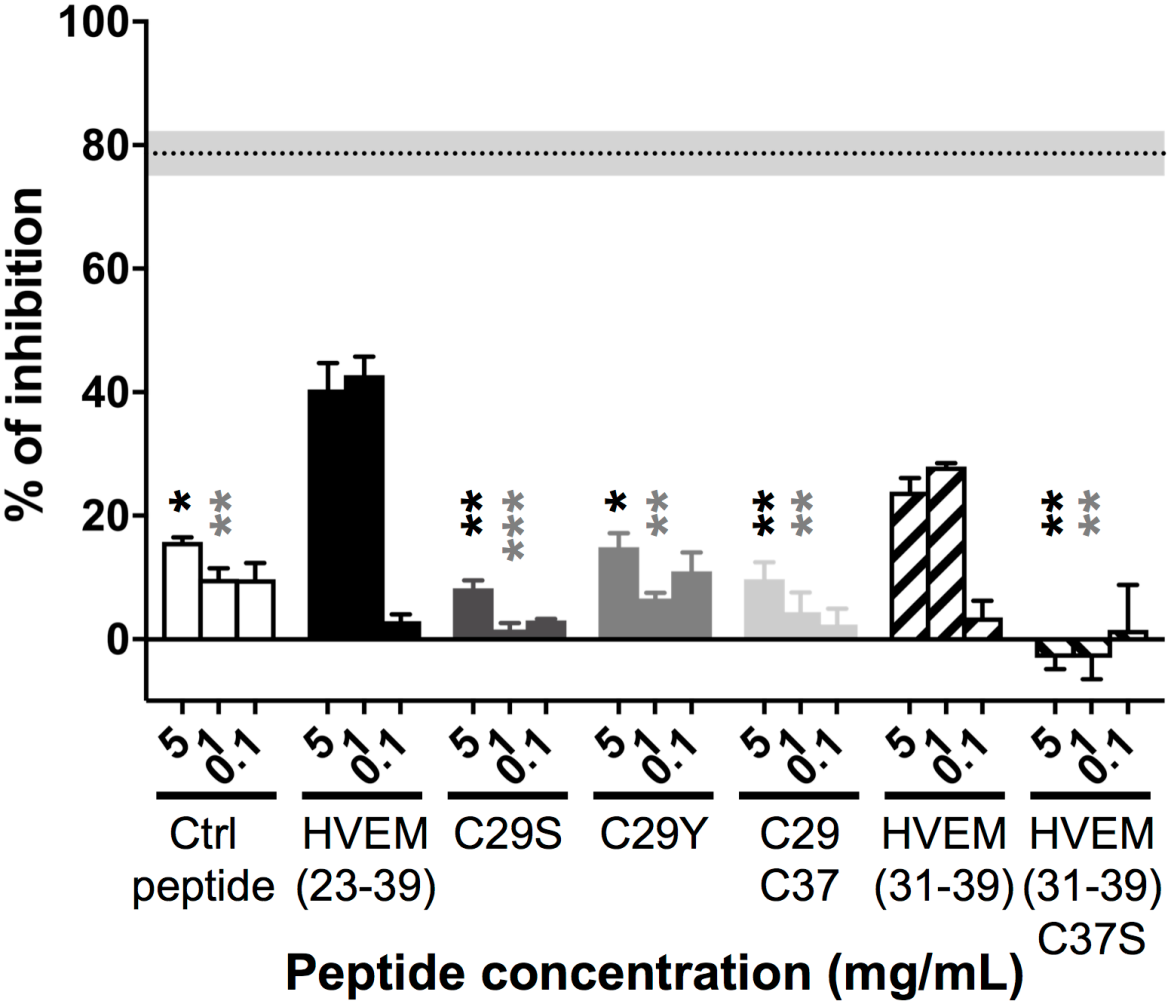
Cysteines from HVEM(23–39) are the main amino acid residues involved in the inhibition of BTLA/HVEM interaction. Increasing concentrations of HVEM(23–39) or HVEM(31–39) modified peptides, where one or both cysteines were substituted, were tested in ELISA and compared to corresponding wild-type and scrambled (Ctrl peptide) peptides (at least two experiments in triplicate). The percentage of inhibition of the BTLA/HVEM binding was calculated in relation to the negative control (PBS). Statistical analysis was performed by comparing each concentration of each peptide to the same concentration of wild-type peptide HVEM(23–39). **: p< 0.01 following non-parametric One-way ANOVA and Dunn’s post-test.

The analogs (Table 2) were tested in the ELISA tests and compared to the HVEM(23–39) and scrambled peptide (Fig 5). We found that both substitutions of Cys29 significantly abrogated the blocking capacity of the HVEM(23–39) peptide. This could indicate the lack of specific interactions of Cys29 residue and point to the importance of the free sulfhydryl group for the binding of peptides. Moreover, albeit not significant, the shorter synthesized peptide: HVEM(31–39) that lacks the Cys29, showed a lower percentage of inhibition than the HVEM(23–39). To further test the importance of Cys37, we synthesized and tested the its analog (HVEM(31–39) C37S). We found that this peptide had no blocking capacity. Finally, the cyclic peptide HVEM(23–39) (C29-C37), encompassing blocked sulfhydryl groups, was also tested, but no blocking of BTLA/HVEM interaction was observed (Fig 5). Of note, in CD spectrum, formation of the disulfide bridge in HVEM(23–39) peptide did not significantly change the peptide structure (S1 Fig).

Then those analogs were tested in affinity test. We observed that peptides, which could inhibit the BTLA/HVEM interactions in ELISA tests (Fig 5), were also bound by BTLA protein immobilized in microcolumn (Table 1). The exception was cyclic peptide HVEM(23–39) (C29-C37) which interacted with BTLA in affinity test but in ELISA test its inhibitory effect was not observed. The results suggest that the binding site of cyclic peptides and HVEM(23–39) on the BTLA surface is different.

Overall, our results confirm the prominent role of free cysteine residues in the BTLA/HVEM interaction blocking capacity of the HVEM(23–39) peptide.

**Fig 6:**
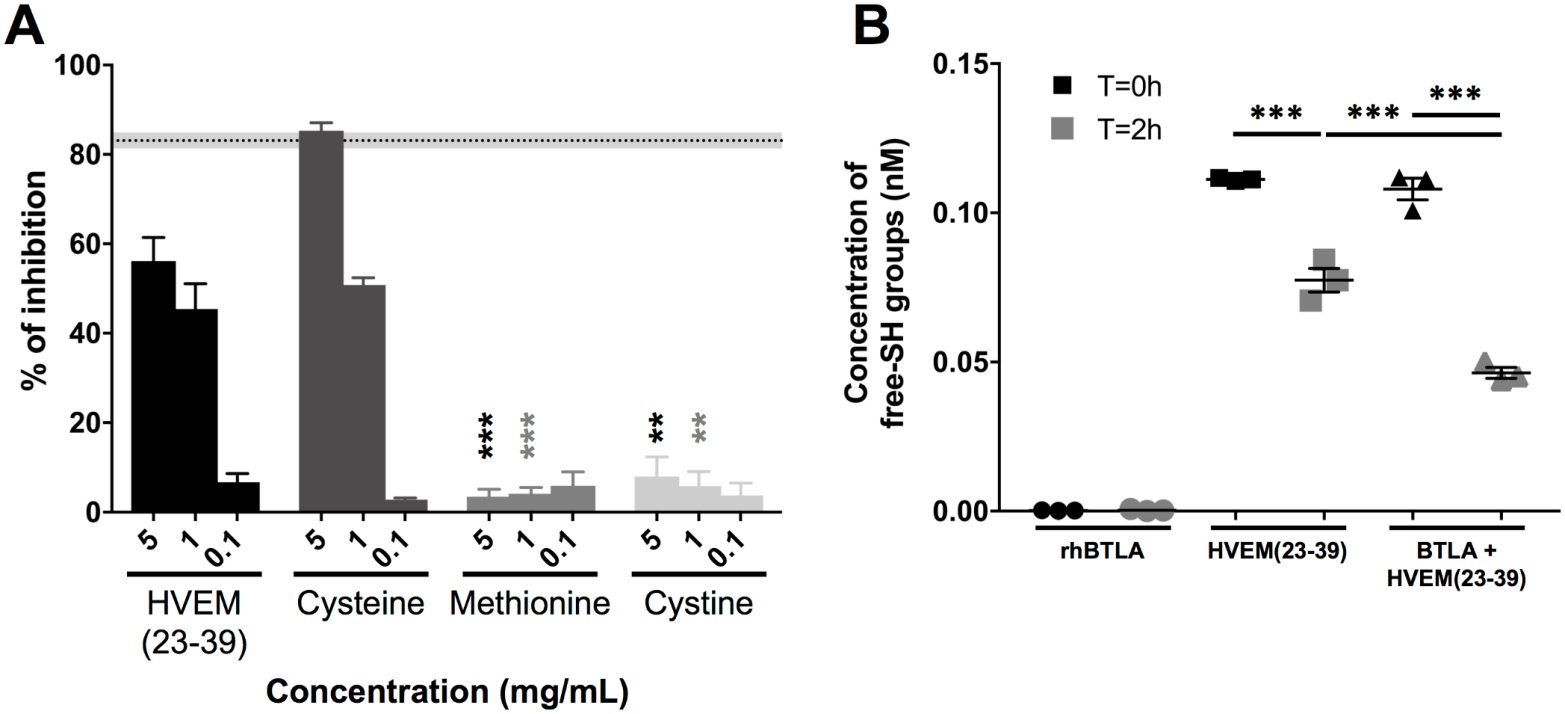
Free cysteine blocks the ligation between BTLA and HVEM. **(A)** The effect of increasing concentrations (5, 1 and 0.1 mg/mL) of free amino acids were tested in BTLA/HVEM ELISA (three experiments in triplicate). The percentage of inhibition was calculated in relation to the negative control (PBS). The gray and dotted back lines correspond to the percentages of inhibition observed with an anti-BTLA blocking antibody (Mean +/-SEM). Statistical analysis was performed by comparing each concentration of residue or peptide to the same concentration of Cys. **(B)** Assessment of free thiol groups over time in rhBTLA-Fc protein and HVEM(23–39) peptide alone or mixed together. **: p< 0.01 and ***: p< 0.001 following One-way ANOVA and Dunn’s **(A)** or Dunnett’s **(B)** post-test.

Since the cyclic peptide C29-C37, where cysteine residues were oxidized, does not block the BTLA/HVEM binding, we wondered whether the blocking ability of HVEM(23–39) fragment may be due to the capacity of HVEM-based peptide cysteines to reduce the cysteine disulfide bridges present on the BTLA surface, or possibly make disulfide bridges with these BTLA cysteines instead. Therefore, the effect of free cysteine and methionine amino acids or oxidized dimer of cysteine (cystine) on the BTLA-HVEM binding was analyzed. Results showed that cysteine with free sulfhydryl group targeted BTLA protein and prevented partially the binding of HVEM, whereas for the methionine and cystine, the inhibitory properties were not observed (Fig 6A). An Ellman’s assay was then performed on both HVEM(23–39) peptide and recombinant BTLA-Fc in order to quantify free thiol groups. In contrast to BTLA-Fc, we observed free thiol groups in the HVEM(23–39) peptide. However, after 2 hours of incubation, the concentration of free thiol groups in HVEM(23–39) peptide was lower, suggesting that a fraction of the peptide is oxidized. Besides, when the HVEM(23–39) peptide was incubated with BTLA-Fc, we also observed a decrease in the concentration of free thiol groups over time, which was more pronounced than in the condition where the peptide was alone (Fig 6B). These results suggest that BTLA protein may form disulfide bonds with the HVEM(23–39) peptide, lowering the amount of free thiol groups in the peptide.

**Fig 7:**
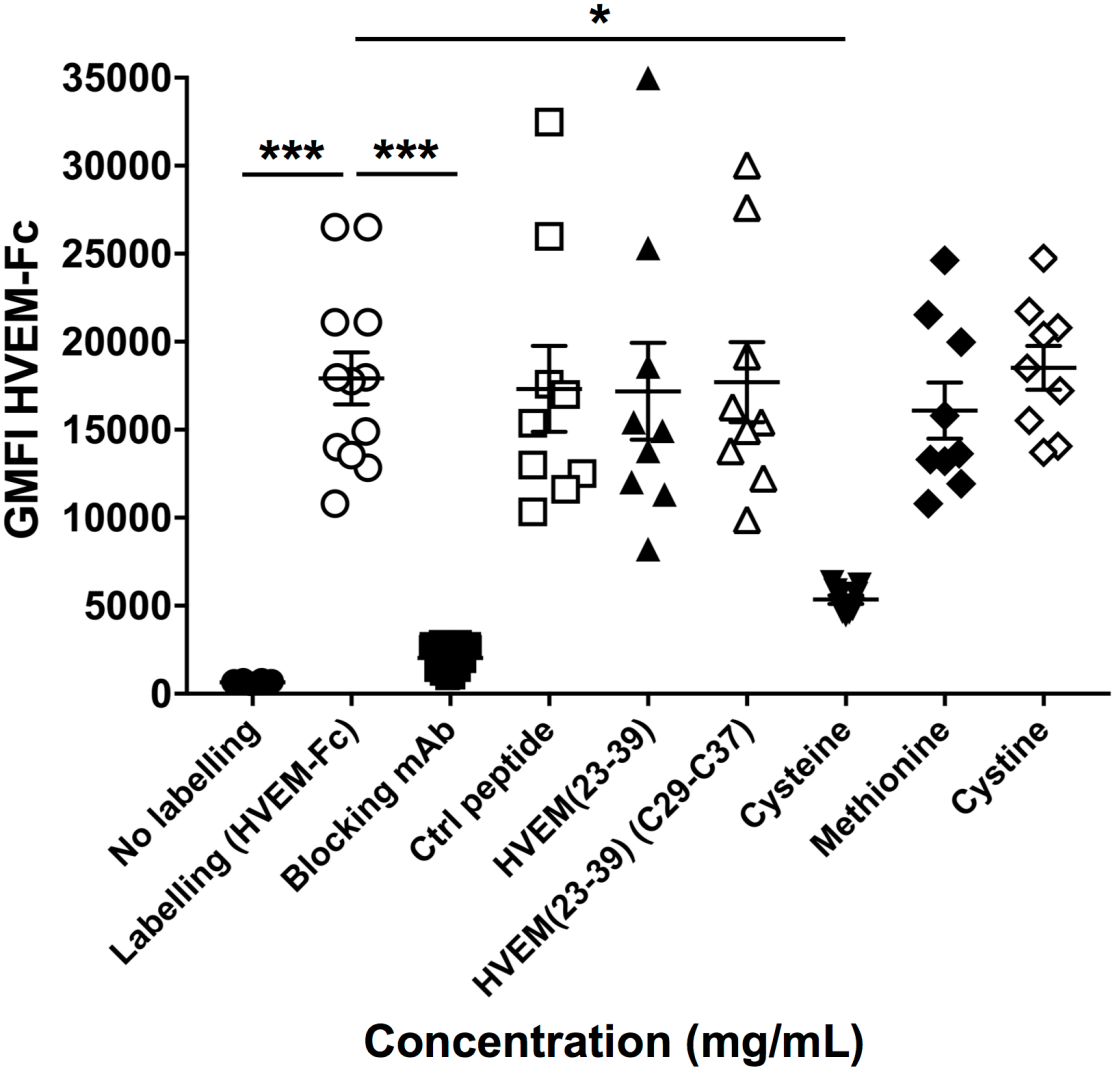
Blocking capacity of HVEM(23–39) fragment and free amino acids in a cellular assay. 293T cells expressing human BTLA were incubated with peptides or free amino acids (5mg/mL) prior labeling with rhHVEM-Fc and AF647-conjugated anti-human IgG antibody (at least two experiments in triplicate). The graph shows the Geometric Fluorescence Intensity (GMFI). *: p< 0.05 and ***: p< 0.001 following non-parametric One-way ANOVA and Dunn’s post-test.

In order to be in a more physiological setting, we developed a cellular assay, where 293T cells expressing BTLA were stained with rhHVEM-Fc followed by AF647-conjugated anti-human IgG antibody. Using this assay, we tested HVEM(23–39) fragment as well as HVEM(23–39) (C29-C37), scrambled peptide (Ctrl), cysteine and methionine amino acids. Surprisingly, in contrast to ELISA tests, almost no blocking of the BTLA/HVEM binding was observed with HVEM(23–39) peptides. However, the free cysteine amino acid blocked BTLA/HVEM complex formation (Fig 7).

## Discussion

Recently, activatory and inhibitory immune receptors and their ligands emerged as promising targets for cancer immunotherapies. Indeed, treatment based on the blockade of immune checkpoints such as PD-1 or CTLA-4, have demonstrated major clinical benefit for cancer patients [40–42]. It is known that BTLA is involved in the negative regulation of T-cell responses by interacting with HVEM [27, 43], highlighting BTLA as a potential target for cancer immunotherapy. Our study focused on the design of blocking peptides of the interaction between BTLA and HVEM, based on the amino acid sequence of HVEM binding fragment. The crystal structure of BTLA/HVEM complex indicated that in the interaction between the two proteins, two fragments of BTLA: (35–43) and (118–128) bind with HVEM: (26–33) and (33–38) respectively. Therefore, HVEM(23–39) fragment was synthesized and its interaction between BTLA and HVEM(23–39) was confirmed by using affinity chromatography. The competitive ELISA tests showed also that the peptide could inhibit the proteins binding. However, we showed using competitive ELISA and cellular assay that this blocking capacity was mainly due to cysteine residues present in the peptide sequence or in solution. Compaan and co-workers showed that the interactions between BTLA and HVEM proteins are primarily stabilized by main chain hydrogen bonds and includes relatively few side chain contacts [30]. The conformation of the HVEM(23–39) peptide is significantly different than the conformation of the same fragment in the native protein due to the fact that it is not stabilized by disulfide bridges and the peptide poses two cysteine residues with free sulfhydryl groups. In the monomeric crystal structure of the extracellular domain of BTLA protein, six cysteine residues form three disulfide bonds between residues 72–79, 34–63, and 58–115. The Cys58–Cys115 disulfide bond is completely buried (solvent accessible surface is 0% for both cysteine) in the hydrophobic core of BTLA [30]. The Cys58–Cys115 (solvent accessible surface is 2,9% and 17,9%) and more Cys34–Cys63 (solvent accessible surface is 45,1% and 33,3%) disulfide bonds are exposed to the solvent and could be sensitive for the other, free or high reactive sulfhydryl group. Therefore, we propose that free sulfhydryl groups of HVEM(23–39) peptide or free cysteine amino acid compete, in our in vitro assays, with disulfide bridges (Cys58–Cys115 or Cys34–Cys63) of the BTLA protein to form covalent S-S bond between HVEM(23–39) peptide or cysteine amino acid and BTLA protein. This may most likely change the structure of BTLA, thus hindering the binding of HVEM. Yet, we observed that the scrambled peptide, which also contains free thiol groups as confirmed by Ellman’s assay (S2 Fig), lacks blocking activity. In order to reduce the disulfide bonds on BTLA, peptides have to pre-dock to the surface of the protein. We hypothesize that scrambled peptide may have a lower affinity to BTLA, compared to HVEM(23–39) peptide. As a consequence of the weaker binding to BTLA the scrambled peptide may not be able to reduce the exposed disulfide bonds of BTLA and form disulfide bonds with the protein.

Finally, the fact that this phenomenon was not observed in the cellular assay might be caused by a spatial arrangement of the BTLA protein on the cell surface making the disulfide bridges on the BTLA surface less accessible for the tested peptides. We hypothesize that the small, compared with peptides, cysteine amino acid could reach the BTLA surface despite the spatial hindrance, hence only activity of cysteine amino acid was observed in the cellular test.

Overall, these data highlight a cysteine-related artefact in vitro, which should be taken in consideration when designing blocking compounds targeting BTLA/HVEM interaction.

## Supporting information

S1 Fig Circular dichroism spectra of HVEM(23–39) and HVEM(23–39) (C29-C37) (A) in water and (B) in phosphate buffer (PBS), pH 7.4 (PDF)

S2 Fig Presence of free thiol groups in HVEM(23–39), scrambled and HVEM(23–39) (C29-C37) peptides assessed by Ellman’s assay. (PDF)

S1 Table Analysis of hydrogen bonds found in the structures of HVEM(23–39)-BTLA complex saved during molecular dynamics simulation. (PDF)

## Abbreviations

APC: antigen-presenting cell
BTLA: B- and T-lymphocyte attenuator
CRD1: cysteine reach domain 1
CTLA-4: cytotoxic T-lymphocyte-associated protein 4
DTT: dithiothreitol
ELISA: enzyme-linked immunosorbent assay
HVEM: herpes virus entry mediator
Ig-superfamily: immunoglobulin-like superfamily
PBS: phosphate-buffered saline
PD-1: programmed cell death 1
RMSD: Root Mean Squared Deviation
TFA: trifluoroacetic acid
TNFR: tumor necrosis factor receptor
