# Supplementary figures and images for: Design of short peptides to block BTLA/HVEM interactions for promoting anticancer T-cell responses
Source: PLoS One. 2017 Jun 8;12(6):e0179201. doi: 10.1371/journal.pone.0179201 (PMC5464627; doi:10.1371/journal.pone.0179201)

A)

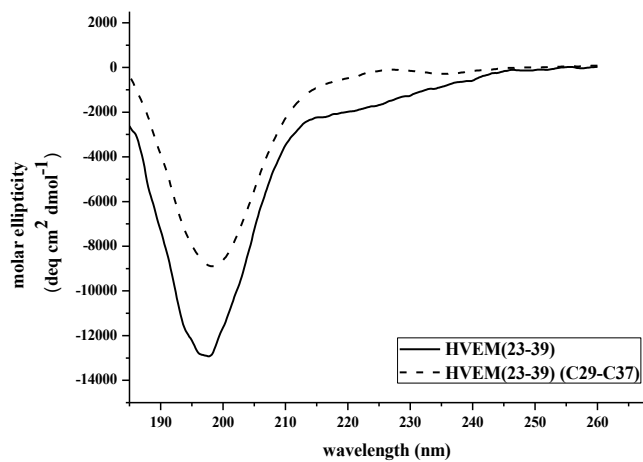

B)

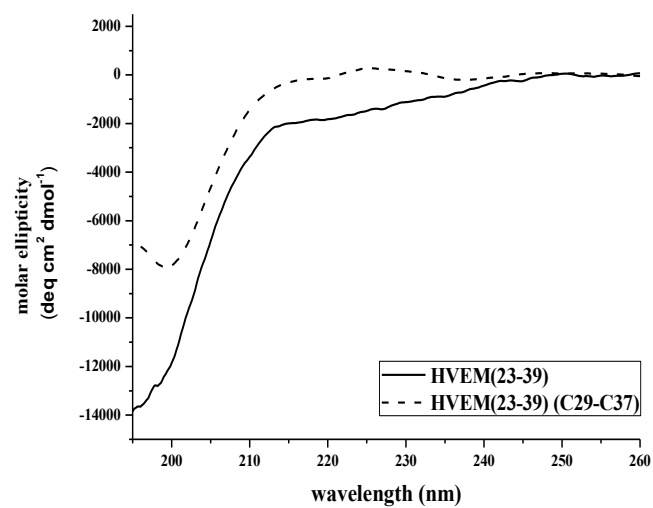

Supplement: S1 Fig — (PDF) [file pone.0179201.s001.pdf]

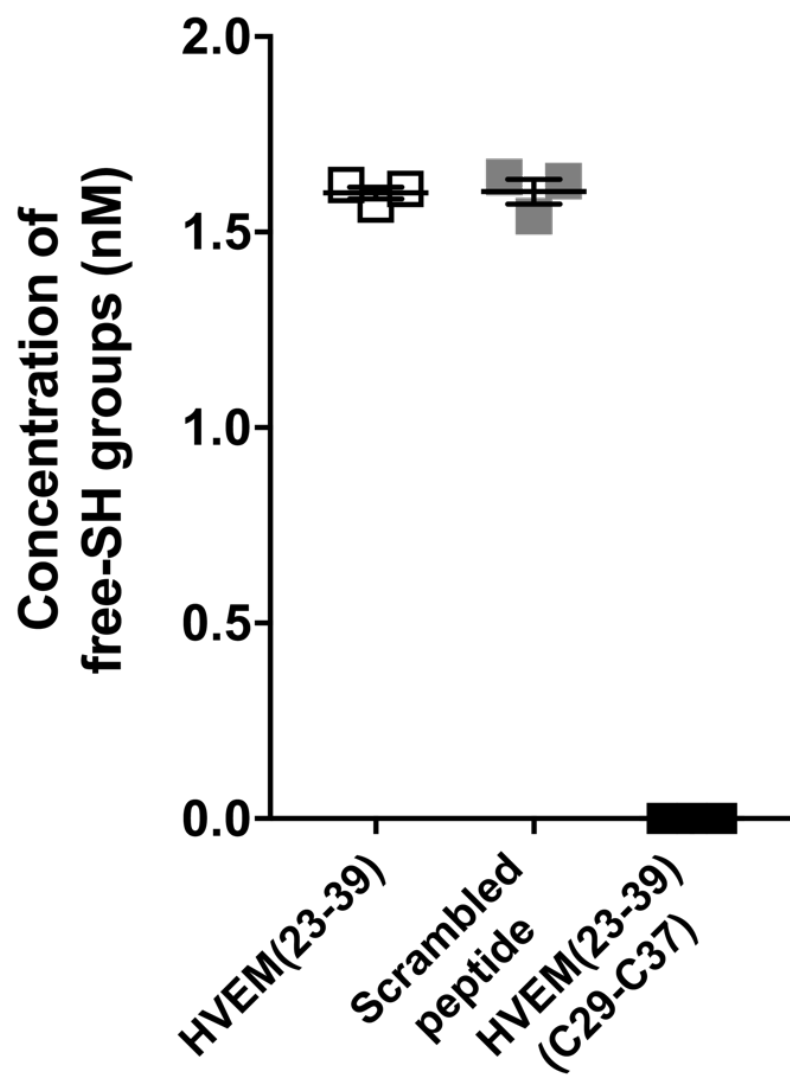

Supplement: S2 Fig — (PDF) [file pone.0179201.s002.pdf]
