# Supplementary material for: Design of short peptides to block BTLA/HVEM interactions for promoting anticancer T-cell responses
Source: PLoS One. 2017 Jun 8;12(6):e0179201. doi: 10.1371/journal.pone.0179201 (PMC5464627; doi:10.1371/journal.pone.0179201)
Supplement: S1 Table — (PDF) [file pone.0179201.s003.pdf]

S1 Table.

Connected residues		% of structures where the bond is found
Arg42 sc	Arg31 sc	51.2%
Arg42 bb	Glu31 bb	96.4%
Glu125 bb	Thr35 bb	96.8%
Leu123 bb	Cys37 bb	96.4%
Gln37 sc	Gly34 bb	84.4%
His127 sc	Gly30 bb	84%
Asn122 sc	Lys26 sc	24%
